# Quantitative Analysis of Inflammatory Uterine Lesions of Pregnant Gilts with Digital Image Analysis Following Experimental PRRSV-1 Infection

**DOI:** 10.3390/ani13050830

**Published:** 2023-02-24

**Authors:** Dávid G. Horváth, Zsolt Abonyi-Tóth, Márton Papp, Attila Marcell Szász, Till Rümenapf, Christian Knecht, Heinrich Kreutzmann, Andrea Ladinig, Gyula Balka

**Affiliations:** 1 Department of Pathology, University of Veterinary Medicine, István u. 2, 1078 Budapest, Hungary; 2 National Laboratory of Infectious Animal Diseases, Antimicrobial Resistance, Veterinary Public Health and Food Chain Safety, University of Veterinary Medicine, István u. 2, 1078 Budapest, Hungary; 3 Department of Biostatistics, University of Veterinary Medicine, István u. 2, 1078 Budapest, Hungary; 4 Centre for Bioinformatics, University of Veterinary Medicine, István u. 2, 1078 Budapest, Hungary; 5 Department of Internal Medicine and Oncology, Semmelweis University, Korányi Sándor u. 2/a, 1083 Budapest, Hungary; 6 Institute of Virology, Department of Pathobiology, University of Veterinary Medicine Vienna, Veterinaerplatz 1, 1210 Vienna, Austria; 7 University Clinic for Swine, Department for Farm Animals and Veterinary Public Health, University of Veterinary Medicine Vienna, Veterinaerplatz 1, 1210 Vienna, Austria

**Keywords:** CD163, digital image analysis, endometrium, inflammatory, PRRSV, QuPath, reproductive

## Abstract

**Simple Summary:**

There is still a huge gap in the knowledge about the reproductive disorders caused by porcine reproductive and respiratory syndrome virus-1. A new, beneficial, and widely used method for the analysis of histopathological lesions in digital image analysis. Using the QuPath software, we aimed to count and classify endometrial inflammatory cells to test the method’s applicability in similar experiments where inflammation needs to be objectively assessed. It proved to be effective and easy to apply, and it would be possible to gain new knowledge about the reproductive disorders caused by porcine reproductive and respiratory syndrome virus-1 by applying similar procedures in the future.

**Abstract:**

Reproductive disorders caused by porcine reproductive and respiratory syndrome virus-1 are not yet fully characterized. We report QuPath-based digital image analysis to count inflammatory cells in 141 routinely, and 35 CD163 immunohistochemically stained endometrial slides of vaccinated or unvaccinated pregnant gilts inoculated with a high or low virulent PRRSV-1 strain. To illustrate the superior statistical feasibility of the numerical data determined by digital cell counting, we defined the association between the number of these cells and endometrial, placental, and fetal features. There was strong concordance between the two manual scorers. Distributions of total cell counts and endometrial and placental qPCR results differed significantly between examiner1’s endometritis grades. Total counts’ distribution differed significantly between groups, except for the two unvaccinated. Higher vasculitis scores were associated with higher endometritis scores, and higher total cell counts were expected with high vasculitis/endometritis scores. Cell number thresholds of endometritis grades were determined. A significant correlation between fetal weights and total counts was shown in unvaccinated groups, and a significant positive correlation was found between these counts and endometrial qPCR results. We revealed significant negative correlations between CD163+ counts and qPCR results of the unvaccinated group infected with the highly virulent strain. Digital image analysis was efficiently applied to assess endometrial inflammation objectively.

## 1. Introduction

Porcine reproductive and respiratory syndrome (PRRS) caused by PRRS virus (PRRSV) is one of the most economically devastating infectious diseases of pigs [1,2]. PRRSV strains belong to the *Nidovirales* order and *Arteriviridae* family [3]. These are small, enveloped viruses with a positive-sense single-stranded RNA genome belonging to two species: PRRSV-1 (*Betaarterivirus suid 1*, formerly European genotype 1) and PRRSV-2 (*Betaarterivirus suid 2*, formerly North American genotype 2). The haptoglobin scavenger receptor CD163 is an essential entry receptor for PRRSV. After the pH-dependent, receptor-mediated endocytosis, the viruses primarily replicate in differentiated alveolar macrophages of pigs (PAMs). Upon knocking out the whole CD163 encoding gene or specifically the receptor binding site (scavenger receptor cysteine-rich domain 5 or SRCR5), pigs and their PAMs become resistant to PRRSV infections [1,4,5,6,7]. Sialoadhesin (Sn), a member of the siglec family of sialic acid-binding molecules, is also involved in the viral entry of PRRSV-1 and can be solely found on specific subsets of differentiated tissue macrophages [8].

Mostly, the viruses cause respiratory disease in growing piglets and fattening pigs and reproductive failure in pregnant sows [2]. The clinical appearance is very diverse; both asymptomatic infection and severe disease may develop, which is influenced, among others, by the virulence of the PRRSV strain and the immunity and genetics of the host [2,9]. In 2006, a highly virulent strain emerged in the Far East characterized by severe symptoms and high mortality in all age groups, and strains of similar virulence have since appeared also in Europe [4,10]. Initially, PRRSV-1 strains belonging to subtype 3, such as Lena and SU1-bel, were considered highly pathogenic; however, more recently, strains from subtypes 1 and 2 were found to cause fatal PRRSV-1 outbreaks. Thus, the “highly virulent” PRRSV-1 strains BOR59 (subtype 2), PR40/2014 (subtype 1), and AUT15-33 (subtype 1) have been reported from 2009 to 2015 in farms from Belarus, Italy, Austria, Germany [10,11,12], and most recently from Spain [13]. While several respiratory models have been published so far, a lot fewer data are available regarding the pathology of reproductive disorders, especially for PRRSV-1 [9,14,15,16].

According to reproductive challenge trials conducted in Canada, PRRSV-2 appears in the endometrium after systemic infection and replicates in CD163+ macrophages. It induces apoptosis in numerous cell types, including macrophages, other inflammatory cells, uterine epithelium, and fetal trophoblasts. Together with the inflammatory lesions, these changes may adversely affect the transfer of nutrients to the fetuses. The viral load of the maternal-fetal interface is a strong predictor of fetal viral load, and its low viral load, together with high fetal thymic viral load, is associated with an increased probability of fetal death [17,18]. According to these findings, viral transmission from maternal to fetal tissues, fetal infection, viral replication in fetal tissues, and viral spread to adjacent fetuses may be cardinal for the development of reproductive disorders in PRRS. Uterine, fetal, and placental factors may also be responsible for disease development [17,18]. Significantly higher viral loads were found in the endometrium, fetal thymus, and fetal serum in the case of meconium-stained fetuses than in other preservation categories in one study, suggesting that these are the most applicable for routine diagnostics [19]. The duration of infection and fetal viral load are probably the leading factors for the prediction of fetal resistance [20].

CD163+ and CD169+ macrophages at the maternal-fetal interface are significantly increased in pregnant gilts infected with PRRSV-2. The viral load of the fetal thymus has a positive correlation with the number of CD163+ macrophages in the endometrium, suggesting the important role of these cells in the pathogenesis of reproductive disorders caused by PRRSV-2 [21]. Macrophages are able to activate certain immune cells, including natural killer (NK) cells; their interaction was already demonstrated in the human uterus. Increased numbers of Sn-positive macrophages and CD8-positive cells were observed in the maternal-fetal interface of sows infected with PRRSV-1. Based on these findings, Sn-positive macrophages can activate endometrial NK cells, which may contribute to the development of histopathological lesions [8].

Anatomically, pigs have incomplete diffuse epitheliochorial placentation without invasion. The maternal and fetal tissues are clearly divided, and the circulation of the mother and the fetus is separated by six tissue layers; therefore, maternal antibodies cannot be transferred during gestation. The embryonic development mostly depends on endometrial gland secretions [15]. Typical histopathological lesions caused by PRRSV infection in the uterus are myometritis and endometritis associated with vasculitis. In the endometrium, the inflammatory infiltrate is predominantly lymphoplasmacytic and histiocytic. If there are fetal lesions, they are varied and not characteristic [9,14]. However, fetal and umbilical lesions were found to be strongly associated with fetal impairment [17].

Digital pathology or virtual microscopy requires the digitization of histopathological slides using whole slide scanners, their storage, and the display and interpretation of the resulting whole slide images (WSI) on a monitor. It has several potential benefits in diagnostics, research, and medical/veterinary education [22,23]. Software capable of digital image analysis (DIA) uses machine learning, meaning that they learn from data and make decisions about new inputs (cells and structures) without human assistance. In the case of supervised learning, the user verifies and modifies the data from which the program can learn [24]. The analyzes performed with these types of software can be more objective and reproducible [25]. The method is increasingly used for the accurate identification and quantification of certain biomarkers, especially in human oncology [26,27]. The variety of colors and shades in routine staining procedures can confuse machine learning techniques and classifications; therefore, in the future, it may be necessary to standardize the entire histopathologic workflow for truly effective digital pathology analyses [28]. However, advanced data-driven algorithms are able to learn a certain degree of image variability [29]. QuPath is an open-access and user-friendly software with various image analysis applications in the field of pathology [30,31]. It is able to classify the detected cells into different categories (classes) using machine learning algorithms in WSIs of hematoxylin and eosin (H&E) stained slides [32].

The aims of our study were to digitally quantify the total inflammatory cell population and the CD163+ macrophages in the endometrial lamina propria of pregnant gilts experimentally infected with PRRSV-1 strains of different virulence. To demonstrate the superior statistical utility of this numerical data compared to manual scoring methods, we also determined the association between the number of these cells and endometrial, placental, and fetal features. The main objective was to determine the validity of this supervised machine learning method in comparison with a semiquantitative method described in a similar experimental setup.

## 2. Materials and Methods

### 2.1. Animal Ethics Approval

The original animal study was reviewed and approved by the Institutional Ethics and Animal Welfare Committee (Vetmeduni Vienna) and the Austrian national authority according to §§ 26ff. of Animal Experiments Act, Tierversuchsgesetz 2012—TVG 2012 (accession number: GZ 68.205/0142-WF/V/3b/2016).

### 2.2. Challenge Experiment

The study material consisted of formalin-fixed and paraffin-embedded (FFPE) blocks of endometrial tissue obtained from an earlier challenge experiment, during which 24 pregnant gilts were infected with a low (720789, 99.76% sequence homology to the PRRSV field isolate IVI-1173 (Genbank Accession number KX622783.1)) or a highly virulent (AUT15-33) PRRSV-1 strain, which has been used for several experimental infection studies in the past [33,34] whereas the low virulent strain also considered causing reproductive pathogenesis in the field, but so far without experimental confirmation [35]. The experimental setup was published in detail by Kreutzmann et al. [36]; briefly, half of the animals were vaccinated with a modified live virus vaccine (Reprocyc PRRS^®^ EU, Boehringer Ingelheim Vetmedica GmbH, Ingelheim am Rhein, Germany) twice before insemination, and then on the 53rd day of gestation according to the manufacturer’s protocol.

The gilts were randomly distributed into six groups: non-vaccinated and non-infected, vaccinated and non-infected, non-vaccinated and infected with the low virulent strain, vaccinated and infected with the low virulent strain, non-vaccinated and infected with the high virulent strain, and vaccinated and infected with the high virulent strain (4 gilts/group). The groups were named no_vacc/no_chall, vacc/no_chall, no_vacc/chall_L, vacc/chall_L, no_vacc/chall_H, and vacc/chall_H, respectively.

**Figure 1:**
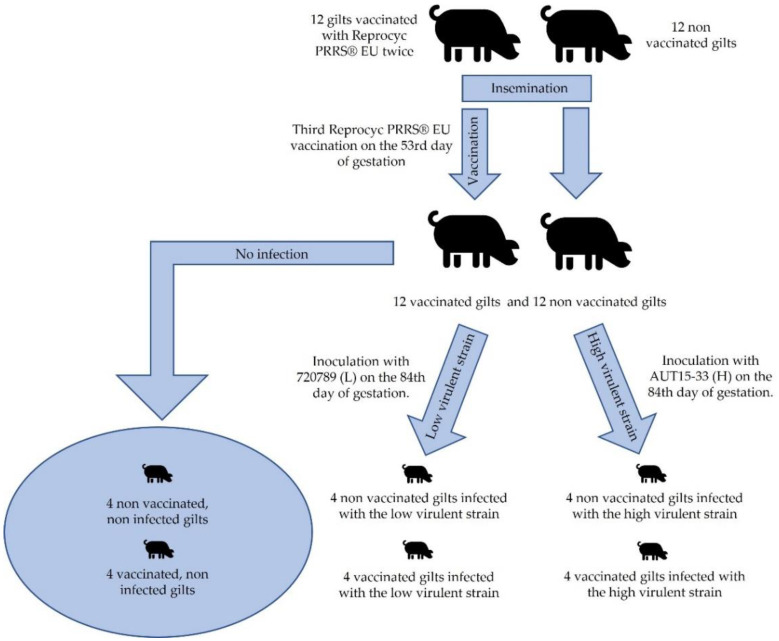
The simplified formation and distribution of the experimental groups.

The experimental design specifically sought to avoid cross-contamination and factors interfering with the evaluation of its outcomes. Four vaccinated and four non-vaccinated gilts were inoculated with 3 × 10^5^ TCID_50_ of either AUT15-33 (H) or 720789 (L) in a total volume of 4 mL; 2 mL were administered intramuscularly, and 1 mL was administered into each nostril on the 84th day of gestation. The simplified experimental setup is shown in Figure 1.

The negative control group was sham inoculated with Dulbecco’s modified Eagle’s medium. At 21 ± 2 dpi, the animals were humanely euthanized. Intravenous ketamine (Narketan^®^ 100 mg/mL, Vetoquinol Österreich GmbH, Vienna, Austria; 10 mg/kg body weight) and azaperone (Stresnil^®^ 40 mg/mL, Elanco GmbH, Cuxhaven, Germany; 1.5 mg/kg body weight) were injected before the intracardiac injection of T61^®^ (embutramide, mebezonium iodide, and tetracaine hydrochloride; Intervet GesmbH, Vienna, Austria; 1 mL/10 kg body weight). All fetuses were individually evaluated and dissected, according to Ladinig et al. [19]. Endometrial samples collected during necropsy were used in our study.

### 2.3. Tissue Processing and Routine Histology

After 24 h of fixation at room temperature in formaldehyde solution, samples were trimmed and dehydrated with a series of ethanol and xylene in an automatic tissue processor. The dehydrated tissue samples were embedded in paraffin blocks, and 4 µm thin sections were cut manually and mounted onto Superfrost+ adhesion slides (Thermo Fisher Scientific, Waltham, MA, USA). The unstained sections were deparaffinized and rehydrated in xylene and alcohol, respectively. Routine H&E staining was performed in an automatic staining instrument.

### 2.4. Section Selection

Our aim was to determine the validity of digital pathology in similar experimental setups by objectively quantifying endometrial inflammatory cells. To illustrate the superior statistical applicability of the obtained numerical data, we also examined associations between digitally quantified total inflammatory cell numbers and manual scorings, CD163+ numbers, PRRSV genome equivalent (GE) values measured by qRT-PCR and the different groups defined by vaccination status and challenge strain virulence.

We have chosen all H&E-stained endometrial slides with a manual endometritis score of at least 1 (*n* = 131): 50 slides were included from gilts that were non-vaccinated and challenged with the low virulent strain (“no_vacc/chall_L”), 45 slides from non-vaccinated gilts challenged with high virulent strain (“no_vacc/chall_H”), 21 slides originated from vaccinated gilts challenged with the high virulent strain (“vacc/chall_H”) and 15 from vaccinated cases infected with the low virulent strain (belonged to the “vacc/chall_L” group).

We used another 10 sections that scored 0 for both endometritis and vasculitis for the calculations performed with the manual scorings. The sections originated from non-infected and infected gilts with no inflammatory lesions in the uterine samples.

The weight of the fetuses from cases with endometrial inflammation ranged from 273 g to 1481 g (mean = 768.2692, median = 793.5, standard deviation = 218.0419, *n* = 130). The manual scoring grades, grouping, weight of the fetuses, and log10 transformed qPCR results are presented in Supplementary Table S1.

### 2.5. CD163 Immunohistochemistry

From the 131 FFPE blocks with inflammation, we randomly selected 35 for CD163 IHC. Rabbit polyclonal antibodies (ab87099, Abcam, Cambridge, MA, USA) were used as primary antibodies against the CD163 glycoprotein. Sections were deparaffinized in xylene and rehydrated in a graded ethanol series. The 15-min endogenous peroxide inhibition (97 mL distilled water + 3 mL H_2_O_2_) was followed by phosphate-buffered saline (PBS) rinsing. Antigen retrieval was performed in citrate buffer (pH 6.0) in a microwave (800 Watts for 5 min, then 180 Watts for 10 min) then the sections were flushed with PBS. We incubated the slides in 2% porcine albumin (Albumin from porcine serum, Sigma Aldrich) for 1 h in a moist chamber; then, without flushing, the primary antibody was added and incubated at a 1:500 dilution in a wet chamber for 30 min. The slides were rinsed in PBS before and after the 20 min incubation of the secondary antibody (Dako, EnVision Flex HRP, Agilent Technologies, Santa Clara, CA, USA). The staining was displayed with DAB Chromogen (Dako Envision Flex DAB + Chromogen 1 drop + 1 mL Envision Flex Substrate Buffer) for 3–4 min, and thereafter the sections were rinsed in PBS. The slides were counterstained with hematoxylin according to GILL II for 30 s; bluing was performed in PBS. After dehydration in ethanol and xylene, the slides were covered with coverslips. Negative tissue control samples were stained without adding the primary antibody, whereas the positive control was a fetal lymph node sample.

### 2.6. Manual Scoring

Inflammation of the endometrium was assessed by two blinded examiners independently according to the method described by Novakovic et al. [9]. Briefly, the severity of the endometrial inflammation (0–4) and vasculitis (0–3) was scored. A 1.1 mm and a 0.55 mm field of view were used for 200× and for 400× magnification, respectively.

In the case of endometritis, the method determines the percentage of the area infiltrated with inflammatory cells and the distribution of the lesions at a 200× magnification. In the case of grade 0, inflammatory cells were rarely present; in grade 1, inflammatory cells were multifocally present in less than 10% of the slide; in grade 2, multifocal to coalescing inflammatory cell infiltration could be identified in 10–25% of the slide, in grade 3 a diffuse inflammatory cell infiltrate appeared in 25–50% of the slide, and in grade 4 inflammatory cells were diffusely present in more than 50% of the slide.

In the case of vasculitis, three areas of 200× magnification were randomly selected, and three blood vessels were evaluated at a 400× magnification based on the inflammatory cell infiltration of their walls and/or their level of degeneration. In grade 1, inflammatory cells appeared within the blood vessel wall; in grade 2, besides inflammatory cells, the degeneration (vacuolation and splitting of smooth muscles) or necrosis of the blood vessel wall could be identified, and in grade 3, the above-mentioned lesions were present simultaneously. A single vasculitis severity score was obtained by averaging the scores of the above-mentioned nine vessels.

### 2.7. Section Scanning and Software Analysis

**Figure 2:**
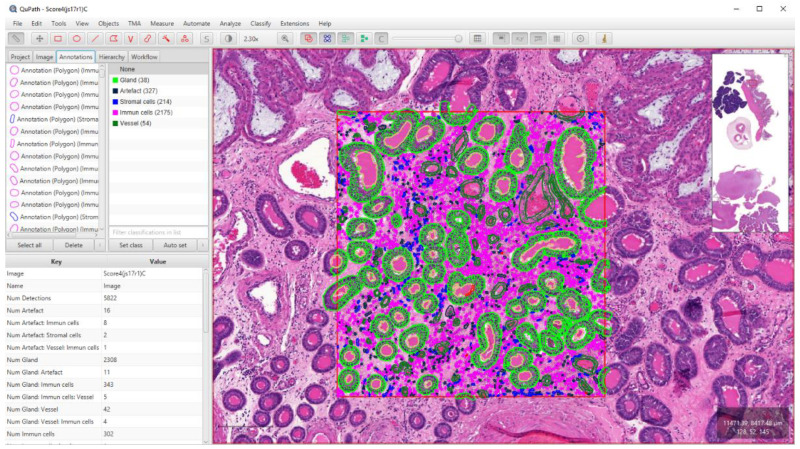
Digital quantification of total inflammatory cells with QuPath software. To train the object classifiers, we manually set the detected cells into the corresponding classes in a 1 mm^2^ area.

The slides were digitized with a Pannoramic Midi slide scanner using a 20× objective (3D Histech, Budapest, Hungary). Whole slide images were further analyzed with QuPath (version 0.2.3.) [30]. We applied the “estimate stain vectors” command in each slide to refine the hematoxylin and DAB stain estimates. We made annotations of 2.37 mm^2^ in the endometrial lamina propria of the H&E-stained slides, which is equivalent to 10 high power field (HPF) areas of conventional light microscopy [37]. On contrary to the manual method, vasculitis was not scored separately; its inflammatory cells were quantified together with the selected propria area. The cell-poor, almost cell-free propria layer of healthy maternal endometrium helped the cell quantification even in the case of routine, H&E-stained slides. The “cell detection” command was selected, then the supervised machine learning method was applied using the “random trees classifier” method within the “train object classifier” command. Altogether 15 distinct classifiers were trained and used in the subsequent analyzes. Each one was trained on a 1 mm^2^ area of slides with distinct color shades and quality. This was necessary due to diverse section quality and color features. All measurements (41 morphological features) were computed for each cell and used as input for classification. Five classes were set to each: “immune cells”, “stromal cells”, “vessels”, “glands”, and “artifacts” (Figure 2).

**Figure 3:**
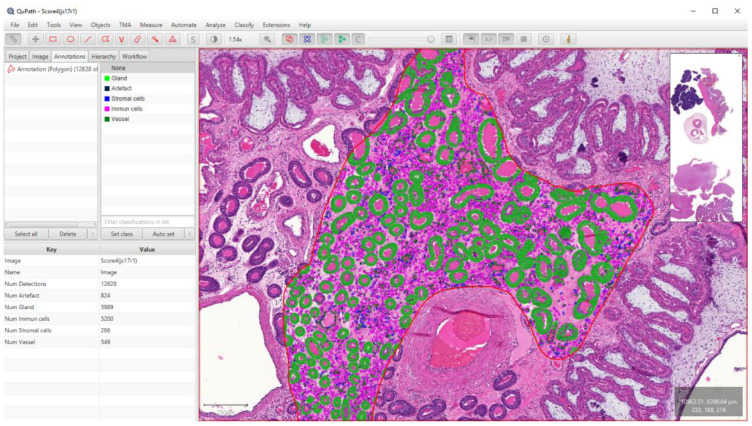
Digital quantification of total inflammatory cells with QuPath software. After the implementation of the cell detection command in a 2.37 mm^2^ area, one of the previously created object classifiers was loaded. If the classifying results were unsatisfactory, we trained a new one based on that image.

**Figure 4:**
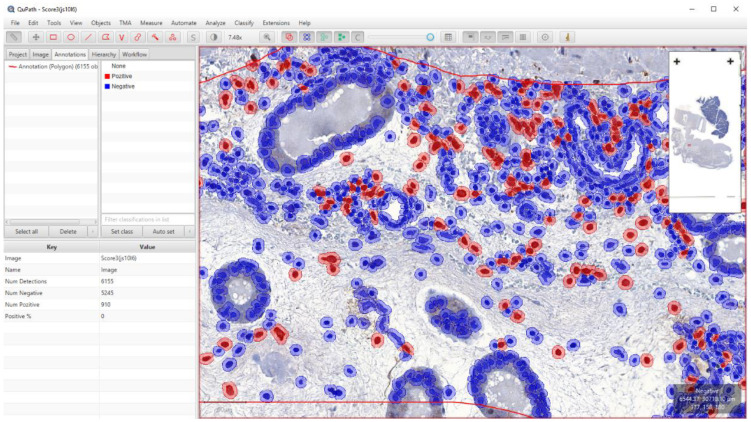
Digital quantification of CD163+ macrophages with QuPath software. In the case of CD163 IHC, we applied the single measurement classification, which was considered the most reliable method for identifying positive macrophages. The DAB-labeled brown macrophages appear red after the classification.

The use of the artifact class was necessary as the cell detection command also identified several non-cellular structures that interfered with the final numbering of the cells. For the slides labeled with CD163 IHC, the methodology was the same until cell detection. After that, we created six single measurement classifiers using “DAB channel filter” and “Cell: DAB OD std dev” as measurements. For each WSI, the different classifiers were evaluated and compared, and the most accurate was selected (Figure 3 and Figure 4). The data were exported to separate Excel sheets (.xlsx) according to the identifier of the case.

A detailed step-by-step description of the processes is provided in Supplementary File S2.

### 2.8. PRRSV qRT-PCR

The qRT-PCR assay to detect and quantify the PRRSV genome was performed on fetal serum and thymus and on endometrial and placental tissues.

PRRSV detecting qRT-PCR was carried out as described by Kreutzmann et al., 2022 [36]. Briefly, tissue and organ sections (50 mg) were homogenized in 600 μL Qiazol (QIAGEN) in a TissueLyser II instrument (QIAGEN) for 3 min. After a quick spin, 300 μL of chloroform was added, and the mixture was thoroughly vortexed. Subsequently, the tubes were centrifuged at 13,000× *g* for 5 min. 200 µL of the aqueous phase was further processed using the cador Pathogen Kit in a QiaCubeHT instrument (QIAGEN) according to the manufacturer’s protocol.

ORF7-specific RT-qPCR was carried out using the Luna Onestep RT PCR Kit (New England Biolabs). The primer sequences were adapted from Egli et al. [38] to fit the sequence of PRRSV-1 strains AUT15-33 and 720789 [39]. As nucleic acid extraction control, porcine β-actin mRNA was quantified by qPCR in all samples according to Toussaint et al. [40].

The absolute quantity of the genome equivalents (GE) was calculated from serially diluted SP6 transcripts of cloned AUT15-33 cDNA fragment 13261–3′ end in a pGEM-T (Promega GmbH, Walldorf, Germany) plasmid (pLS69) [39]. Quantitative RT-PCR was done in an Applied Biosystem 7300 instrument (Applied Biosystems, Thermo Fisher Scientific Inc., Waltham, MA, USA).

### 2.9. Statistical Analyzes

For statistical evaluation, we used the R program (4.1.1) (R Foundation for Statistical Computing, Vienna, Austria). The concordance of the two examiners’ scorings was compared using Fleiss’ kappa. We compared the distributions of inflammatory cell counts (total and CD163+ cells) between the groups and examiner1’s manual endometritis scoring grades, and the distributions of log_10_ transformed qPCR-results between the same grades by Kruskal–Wallis test with Holm correction (alpha = 0.05). This method was selected due to unsatisfactory normality conditions. The correlation between endometritis, vasculitis scores, and digitally quantified inflammatory cell numbers were examined by Kendall correlation.

The concordance between the total cell count and examiner1’s endometrial inflammation scoring was calculated by Fleiss’ kappa. Thresholds between the five endometritis intervals (0–4) were calculated with 1000 simulations, considering a randomly selected 2/3 of the data and validating the results on the remaining 1/3. For demonstrative purposes, we used plots with different bandwidths, with the x-axis showing the cell number and the y-axis showing the proportion of examiner1’s endometritis scores in the (x ± bandwidth) range of total cell counts. Pearson correlation coefficient was used to evaluate the correlation between inflammatory cell number and fetal weight, as well as between inflammatory cell count and GE PRRSV values measured by qRT-PCR.

The data analyzed in this study are available in Supplementary Table S1.

## 3. Results

### 3.1. Comparison of the Manual Scorers

In the case of vasculitis severity, an equal decision was made in 79.4% of all observations between the two examiners, the Fleiss’ kappa was 0.68 (*p* < 0.0001). There was a greater agreement among the examiners in the case of endometritis, where the frequency of the same decision was 90.0%, and the Fleiss’ kappa was 0.85 (*p* < 0.0001). The results indicate a strong concordance between the results given by the two examiners.

### 3.2. Section Scanning and Software Analysis

Altogether 141 H&E-stained and 35 CD163 IHC-labeled histopathological slides were digitalized and analyzed. The inflammatory cell numbers of each slide with the associated data are shown in Supplementary Table S1.

The total number of inflammatory cells calculated on 2.37 mm^2^ areas ranged from 92 to 5200 (mean = 1279.94, median = 798.0, standard deviation = 1205.64, *n* = 141). Similarly, the number of CD163+ macrophages ranged from 38 to 910 (mean = 177.83, median= 127, standard deviation= 156.39, *n* = 35). Numerical summaries of total inflammatory cell counts in examiner1’s endometritis grades and within the infected groups are seen in Table 1 and Table 2.

### 3.3. Distributions of Cell Counts and qPCR-Results

**Figure 5:**
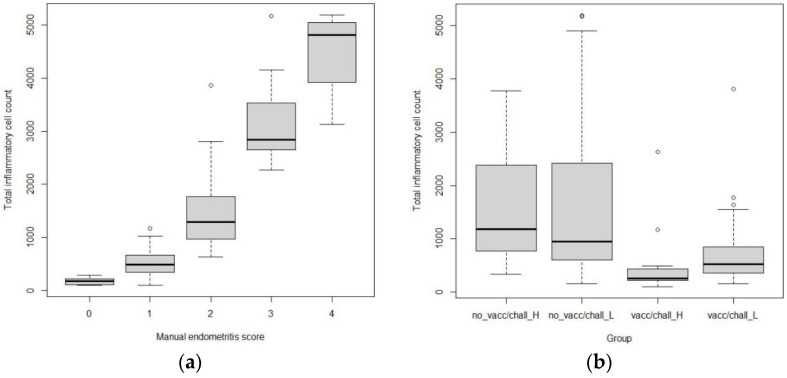
Distributions of total inflammatory cell counts. The boxplots of total inflammatory cell counts show the separation of examiner1’s manual endometritis grades (**a**) and the markedly different distributions of total inflammatory cell counts between the vaccinated and unvaccinated groups (**b**). The circles above the bars are individual cases with substantially higher cell numbers.

**Figure 6:**
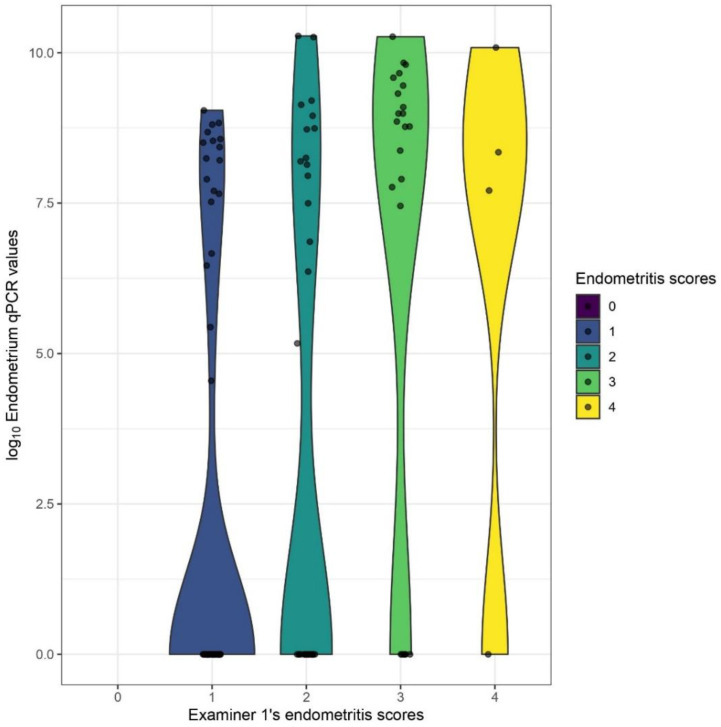
Violin plots depicting the distributional differences of log10 endometrial qPCR values between different endometritis grades as evaluated by examiner1.

**Figure 7:**
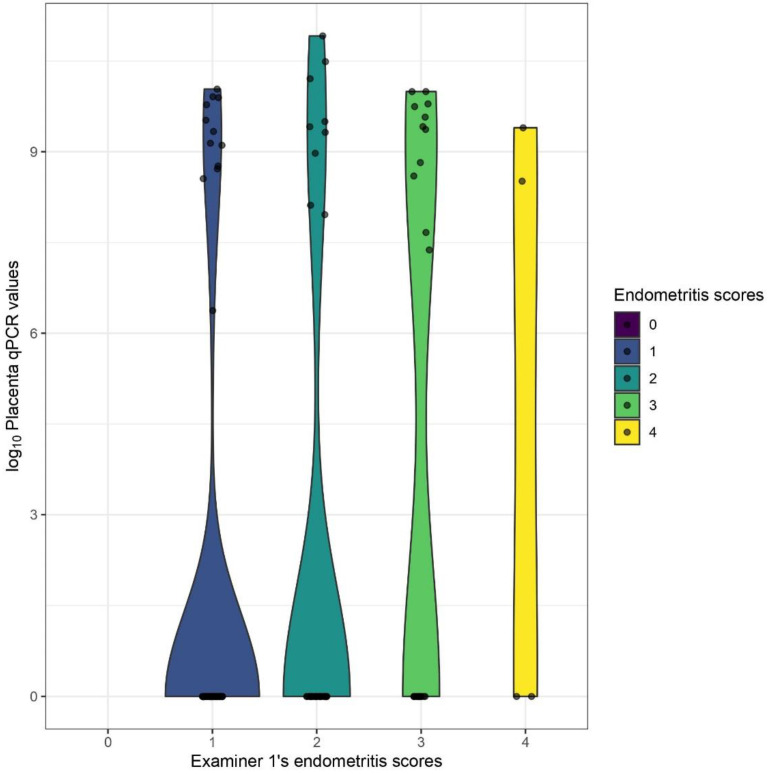
Violin plots depicting the distributional differences of log10 placental qPCR values between different endometritis grades as evaluated by examiner1.

The distributions of total inflammatory cell counts differed significantly between each manual endometritis grade of examiner1 (*p* < 0.0001), although remarkable overlaps were found between the different levels (Figure 5a). The difference was significant between the groups as well (*p* < 0.0001), except between the two non-vaccinated ones (Figure 5b). The distribution of CD163+ cell counts showed no statistically significant difference between examiner1’s manual endometritis grades or between the different groups. The distributions of endometrial and placental qPCR GE values differed significantly between examiner1’s manual endometritis grades (*p* = 0.0001 and 0.039) (Figure 6 and Figure 7).

### 3.4. Associations of Cell Counts and Manual Scores

Kendall correlations show that regardless of the examiner, a higher vasculitis score is associated with a higher endometritis score, and a higher total inflammatory cell count is also expected with a high vasculitis or endometritis score (tau = 0.68–0.78, *p* < 0.0001). No statistically significant associations were found between the number of CD163+ cells and total inflammatory cell count or manual scores.

### 3.5. Cell Number Thresholds of Endometritis Grades

**Figure 8:**
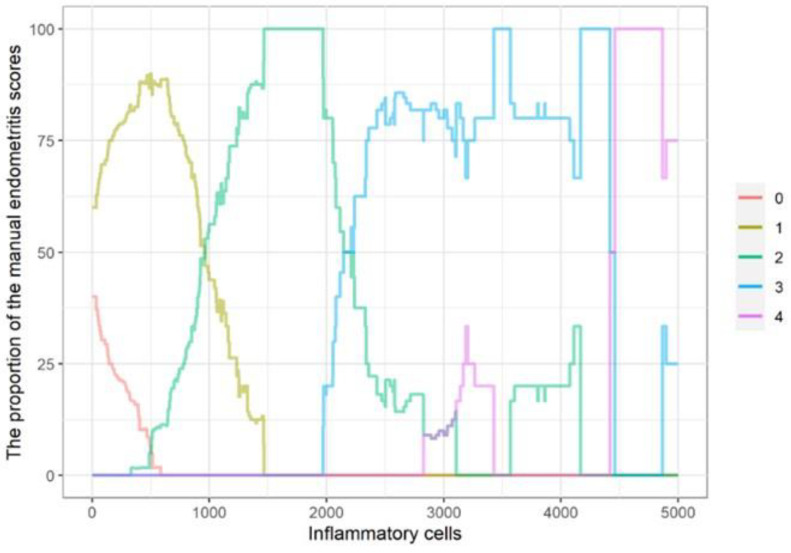
The proportion of examiner1’s manual endometritis scores according to the total cases in the interval of inflammatory cell number ± 300. As the inflammatory cell count increases, the manual endometritis scores also increase, but there is considerable overlap between the different severity categories.

According to simulations, the first threshold (grade 0–1) fell between 95.5 and 161 in 95% of cases, the second (1–2) between 817.5 and 1012, the third (2–3) between 2275 and 2752, and the fourth (3–4) between 3985 and 5038.5. Hit rates ranged from 66% to 87.2% in 95% of cases (Table 3). The proposed thresholds were 105.0 (0–1), 948.5 (1–2), 2398.0 (2–3), and 5038.5 (3–4), in which case there was 78.72% agreement with the manual scoring. A bandwidth of 300 was found to be optimal for the demonstration of total inflammatory cell count thresholds (Figure 8).

### 3.6. Correlations between Cell Counts and Fetal Weights/qPCR-Results

**Figure 9:**
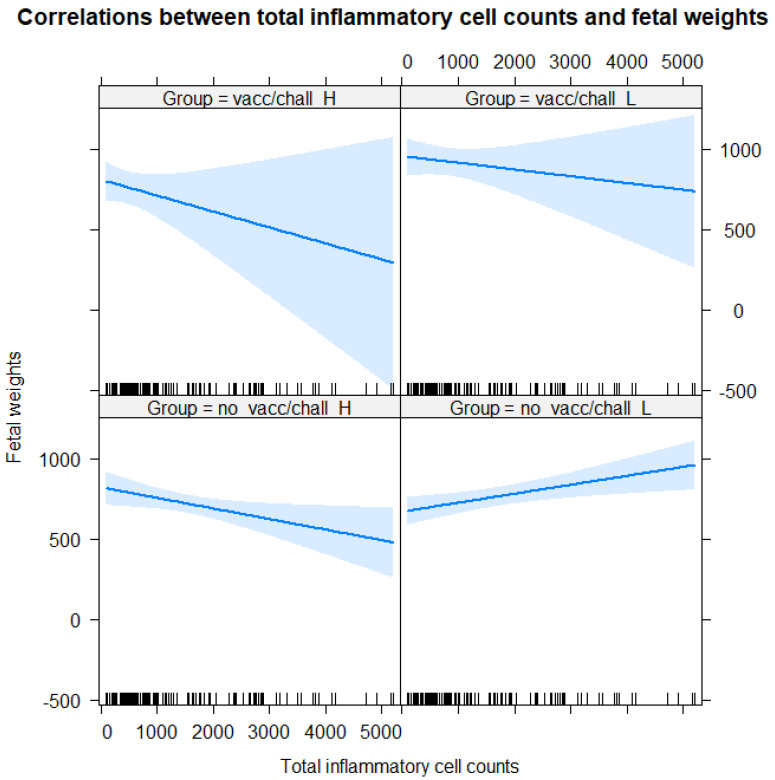
Correlations between fetal weights and total inflammatory cell counts. Based on the width of confidence bands, a significant correlation between fetal weight and total inflammatory cell count only appears in the unvaccinated groups.

**Figure 10:**
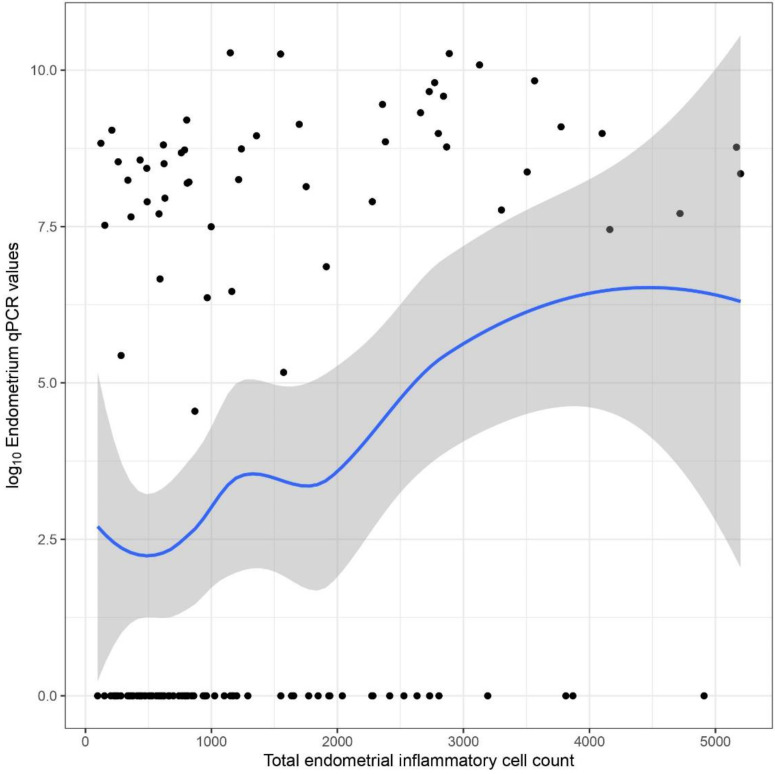
Scatterplot indicating the association between the total inflammatory cell count and endometrial PRRSV GE values.

A significant correlation between fetal weights and total inflammatory cell counts was only found in the unvaccinated groups (Figure 9). In group “no_vacc/chall_L”, there was significant moderate positive (Pearson’s correlation coefficient = 0.34, *p* = 0.0213, 95% CI: 0.05 and 0.58), whereas in the “no_vacc/chall_H” group, there was a significant moderate negative correlation (Pearson’s correlation coefficient = −0.35, *p*= 0.0151, 95% CI: −0.57 and −0.07). A significant overall positive correlation was found between total inflammatory cell counts and endometrial PRRSV GE values (Pearson’s correlation coefficient = 0.32, *p* = 0.0002, 95% CI: 0.16 and 0.47) (Figure 10).

**Figure 11:**
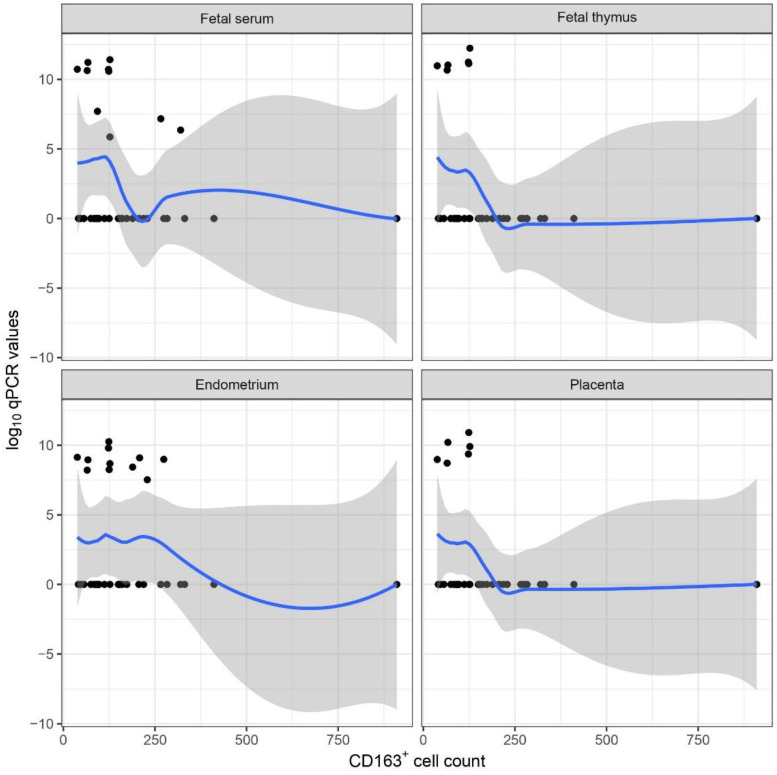
Scatterplot on the association between CD163+ cell counts and log10 qPCR values of the fetal serum, fetal thymus, endometrium, and placenta samples.

No statistically significant correlation was found between CD163+ cell counts and fetal weights; however, we identified statistically significant negative correlations between the number of CD163+ cells and PRRSV GE values of fetal serum, fetal thymus, endometrium and placenta in the “no_vacc/chall_H” group (Pearson’s correlation coefficient = −0.56, −0.72, −0.61, −0.71, *p*= 0.0469, 0.0060, 0.0271, 0.0070, 95% CI: −0.85, −0.91, −0.87, −0.91 and −0.01, −0.27, −0.09, −0.25, respectively) (Figure 11).

## 4. Discussion

The aim of our study was to apply digital image analysis and virtual microscopy on endometrial whole slide images to assess its applicability to replace semiquantitative, more subjective, and less reproducible scoring methods. Total inflammatory cell numbers and CD163+ cell numbers were both quantified and statistically compared to different variables measured during our experiment. Due to the case selection bias, we could not reveal real associations, yet our digital methodology may be useful for more robust future studies using similar variables and statistical analyses.

Digital pathology is widely used in human medicine; however, recently, numerous data have been published about its applicability in veterinary pathology. Bertram et al. [41] compared the diagnostic performance of the evaluation of WSIs and traditional histopathological slides in canine cutaneous tumors without using DIA. There was only a slight difference between the two methods; in the case of round-cell tumors, the digital version had some minor limitations. The mitotic count can vary enormously between areas of canine cutaneous mast cell tumors, and advanced machine learning (deep learning) systems can potentially detect the regions with the highest mitotic count more accurately and reproducibly, even if the current manual grading method is also highly reproducible among pathologists in most cases [42]. Seung et al. [43] analyzed the association between HER2 mRNA expressions of canine mammary tumors and their IHC stainings and quantified the in situ hybridization (ISH) results with open-source image analysis software. A significant correlation was found, and due to the similarities of spontaneous canine mammary tumors to human breast cancers, studies of this nature are also valuable to human research.

Barrera-Zarate et al. [44] used digital pathology for the examination of the porcine maternal-fetal interface. They evaluated the changes in angiogenesis and cell proliferation after PRRSV-2 infection using ImageJ software combined with vascular endothelial growth factor (VEGF) and Ki-67 immunofluorescence. According to the study, fetal resilience may be associated with greater uterine epithelial proliferation and fetal compromise with lower uterine submucosal angiogenesis. Intrauterine growth-restricted fetuses with inherently lower submucosal angiogenesis may be protected to a certain extent against PRRSV-2 infection.

Since its release in 2017, QuPath has also been used for DIA in several studies evaluating animal tissues. QuPath could reliably identify gliotic expansion, axon loss, and morphological changes in degenerating optic nerves of brown Norway rats suggesting its usefulness in estimating therapeutic efficacy in preclinical treatment trials of glaucoma [45]. Finney et al. [46] developed an automated method to quantify reactive astrocytic responses in WSIs of rat brains using glial fibrillary acidic protein (GFAP) and 3,3′-diaminobenzidine (DAB) IHC. QuPath also has the potential to evaluate diffuse brain injury and any kind of disorder having extensive but slight effects on protein expression levels [47]. Last year our research group successfully applied QuPath for the detection and quantification of RNAscope ISH signals of the PRRSV genome in porcine lung tissues infected with PRRSV-1, and a significant association was found between the proportion of the infected cells and the PRRSV GE numbers measured by qPCR in the same lung lobe [48].

At first, we compared the different inflammation scores between the two examiners. A fair agreement was found between the manual scoring points at the applicability of this traditional, semiquantitative method despite its potential subjectivity. As the scoring of grades did not show a significant difference, for further statistical analysis, we used only examiner1’s grades due to his expertise.

Significant differences were found in the distribution of total inflammatory cell numbers between the manual endometritis grades of examiner1, proving that digital image analysis can be used to objectively characterize and even categorize endometrial inflammation comparable to manual scoring. It has to be stressed that there were notable overlaps and standard deviations between the grades, probably because the methods compared are substantially different, and there is no standardized scoring system for porcine endometrial inflammation.

The distribution of total inflammatory cell counts also differed significantly between groups, except between the two non-vaccinated ones. Even though the aim of our study was not the comparison of lesion severity between the strains, unvaccinated animals may have similar inflammatory cell numbers in their endometrium regardless of the viral strain and fetal preservation. In the future, more robust studies are necessary to reveal the true associations between viral strains and inflammatory cell counts.

Total cell number thresholds of different endometritis severity categories can be demonstrated by different bandwidth curves or determined by simulations. Our attempt to grade and determine absolute scales based on cell numbers is indicative only, but with a large number of sections, we would likely be able to develop a more objective digital scoring method that automatically assigns endometritis sections to the appropriate severity category. Manual grading of endometritis may potentially always have a higher error rate than DIAs due to the lack of a standardized, well-established scoring method.

A significant correlation between fetal weights and total inflammatory cell numbers was found only in the non-vaccinated groups. Interestingly, the correlation coefficient showed a similar value, but it was positive for the “no_vacc/chall_L” group and negative for the “no_vacc/chall_H” group. Most of the slides used in this study came from these unvaccinated groups, so it is conceivable that we could have obtained similarly significant correlations in the vaccinated groups if we had had more samples with inflammation in them. Vaccination most likely had a protective effect on the endometrium as well.

The positive correlation between total endometrial inflammatory cell counts and PRRSV qPCR GE values suggests that higher viral loads in the fetomaternal interface elicit a stronger inflammatory response. The latter was also suggested by a significantly different distribution of endometrial and placental PRRSV GE values between manual endometritis grades of examiner1.

The statistically significant negative correlation between the number of CD163+ cells and PRRSV GE results of the fetal serum, thymus, endometrium, and placenta in the “no_vacc/chall_H” group is in contrast with the result of Novakovic et al. [21]. In their publication, the PRRSV-2 viral load of the fetal thymus was positively related to CD163+ cell counts in the endometrium. As highlighted before, due to the case selection bias, we could not reveal real statistical associations. However, the role of these cells could be more complex, and CD163 IHC supplemented with cell quantification may not be sufficient to analyze the subtle mechanisms of reproductive disorders caused by PRRSV and the differences between the pathogenesis of PRRSV-1 and PRRSV-2 infection.

Our study had some limitations. Mostly slides with lesions were selected, and these were not necessarily representative of the group. The number of slides obtained from the different groups was very uneven, as there were a lot more endometrial inflammatory lesions in the different groups. We had to work with a relatively small number of samples, especially in the case of higher manual grades and IHC slides. The random trees classifiers used to exclude cells of the proprial glands sometimes made errors by adding inflammatory cells to glandular cells in cases of high-grade inflammations. In these cases, manual corrections were applied. Classification of lymphocytes and plasma cells was the most dependable, granulocytes were occasionally evaluated as artifacts, and macrophages with diverse morphologies were sometimes classified as other cell types.

Summarizing our data, it can be concluded that we have successfully applied digital image analysis in endometrial sections of pregnant gilts experimentally infected with PRRSV-1 for the quantification of inflammatory infiltration.

The open-source QuPath software was shown to be an excellent platform for algorithm training and revision. It was capable of detecting and classifying different cells, but the manual correction was often required due to the varied quality and hues of slides. To our knowledge, this is the first study to investigate the use of DIA to assess PRRSV-induced inflammatory lesions in porcine endometrium.

## 5. Conclusions

In conclusion, DIA can be an efficient method for tissue examination in similar research procedures in addition to or instead of semiquantitative scoring. With its methodology, we can gain new knowledge about the reproductive pathology of PRRSV-1. This was the first study that investigated PRRSV-induced inflammatory lesions in porcine endometrium using digital pathology.

## Figures and Tables

**Table 1:** Numerical summaries of total inflammatory cell count according to examiner1’s manual endometritis grades.

Grade	Mean	Standard Deviation	Minimum	Median	Maximum
Grade0 (*n* = 10)	166.6	65.5	92	178	285
Grade1 (*n* = 67)	521.9	249.57	97	490.0	1167
Grade2 (*n* = 37)	1477.57	658.52	631	1289.0	3869
Grade3 (*n* = 23)	3096.13	730.6	2273	2842.0	5167
Grade4 (*n* = 4)	4489.25	928.8	3128	4814.5	5200

**Table 2:** Numerical summaries of total inflammatory cell count according to treatment groups.

Group	Mean	Standard Deviation	Minimum	Median	Maximum
no_vacc/chall_L (*n* = 45)	1669.49	1505.69	154	950	5200
no_vacc/chall_H (*n* = 50)	1577	978.3	337	1184	3775
vacc/chall_L (*n* = 21)	827.95	832.29	151	529	3813
vacc/chall_H (*n* = 15)	496.07	644.35	97	260	2632

**Table 3:** Determining the thresholds of different endometritis severity categories using the digitally quantified total inflammatory cell numbers. The first threshold value indicates the cell count above which we would score grades 1 and 0 below; therefore, the four threshold values would separate the four endometritis grades. Thresholds, *p*-values with extreme values, and confidence intervals based on 1000 simulations. The simulations were used to evaluate sensitivity. Two-thirds of the cases were randomly selected to determine the threshold values; then, the hit rate was tested on the remaining 1/3 of whether there were tendency differences.

	First Threshold	Second Threshold	Third Threshold	Fourth Threshold	Hit Ratio	*p*-Value
Minimum	95.5	776.5	2156	3404	0.5745	0.0001
Maximum	221	1156.5	2803.5	5038.5	0.9362	1
CI 2.5%	95.5	817.5	2275	3985	0.6596	0.0215
CI 97.5%	161	1012	2752	5038.5	0.8723	1

## Supplementary Materials

The following supporting information can be downloaded at: https://www.mdpi.com/article/10.3390/ani13050830/s1; Supplementary Table S1 and Supplementary File S2. Supplementary Table S1 includes the digitally quantified inflammatory cell numbers, the manual scores, groupings, fetal weights and PRRSV GE results. In Supplementary File S2 the detailed digital methodology and the computed morphological features can be found.
