# Supplementary material for: Quantitative Analysis of Inflammatory Uterine Lesions of Pregnant Gilts with Digital Image Analysis Following Experimental PRRSV-1 Infection
Source: Animals (Basel). 2023 Feb 24;13(5):830. doi: 10.3390/ani13050830 (PMC10000175; doi:10.3390/ani13050830)
Supplement: Supplementary file 1 [file animals-13-00830-s001.zip › Supplemental File S2.pdf]

Digital Image Analysis Methodology

1. We made 2 separate projects and added all WSIs, in each of them image type was set to Brightfield (H&E) or Brightfield (H-DAB) in case of CD163 IHC.
2. We applied the estimate stain vectors command in each WSI to refine the hematoxylin and DAB stain estimates.
3. We made annotations of 2,37 mm^2 in the endometrial lamina propria, which is equivalent to 10 high power field (HPF) of conventional light microscopy. Unlike the manual method, the inflammation of the vessels was not considered separately, it was quantified together with the selected propria area. Inflammatory infiltrate of smaller and medium-sized vessels were also included in the examined region accordingly. We tried to select representative regions of interests (ROI) containing the highest density of inflammatory or CD163+ cells and free of larger empty spaces, large vessels, and artefacts. The easily performable cell counting on routine, HE-stained sections was possible due to the cell-poor, almost cell-free propria layer of the healthy maternal placenta.
4. The default cell detection command was performed from the analysis menu, except that the requested pixel size was set to 1 μm.
5. We used supervised machine learning method and trained 15 distinct random trees classifier that performed the classification on the slides. This was necessary due to the very diverse section quality and color features. All measurements (41 morphological features) were computed for each cell and used as input for the classifier. 5 classes were set to each, namely immun cells, stromal cells, vessel, gland, and artefact. The use of artefact class was found necessary because the cell detection command also identified several non-cellular structures that interfered with the final numbering of the cells, and we considered that this was the most reliable way to subtract them from this calculation. Each classifier was trained on a 1 mm^2 area from 15 different slides with distinct color shades and quality. All detected cells of this region were added to the appropriate class.
6. For the CD163 IHC we did not need to train the classifiers after cell detection, we simply had to select the create single measurement classifier within the Classify–object classification tab. Correspondingly due to the diverse slide quality and colors we applied 6 single measurement classifier with different measurement thresholds. The following parameters were used with live preview: object filter, cells; channel filter, DAB; measurement, cell DAB OD std dev; threshold, 0.14, 0.16, 0.20, 0.22, 0.25 and 0.26; above treshold, pozitive; below treshold, negative.
7. These saved classifiers were used threafter in the adequate slides. For each WSI, the classifier that suitably grouped the cells, especially the inflammatory cells, had to be selected, and if the result was unsatisfactory, a new one had to be taught.
8. We saved all of the annotation measurements, but we only dealt further with the most reliably counted and classified immune cells. These data were exported to separate Excel sheets (.xlsx) according to the unique identifier of the animal, together with the manual scores, the group, the qPCR-results, and the weight of the fetuses.

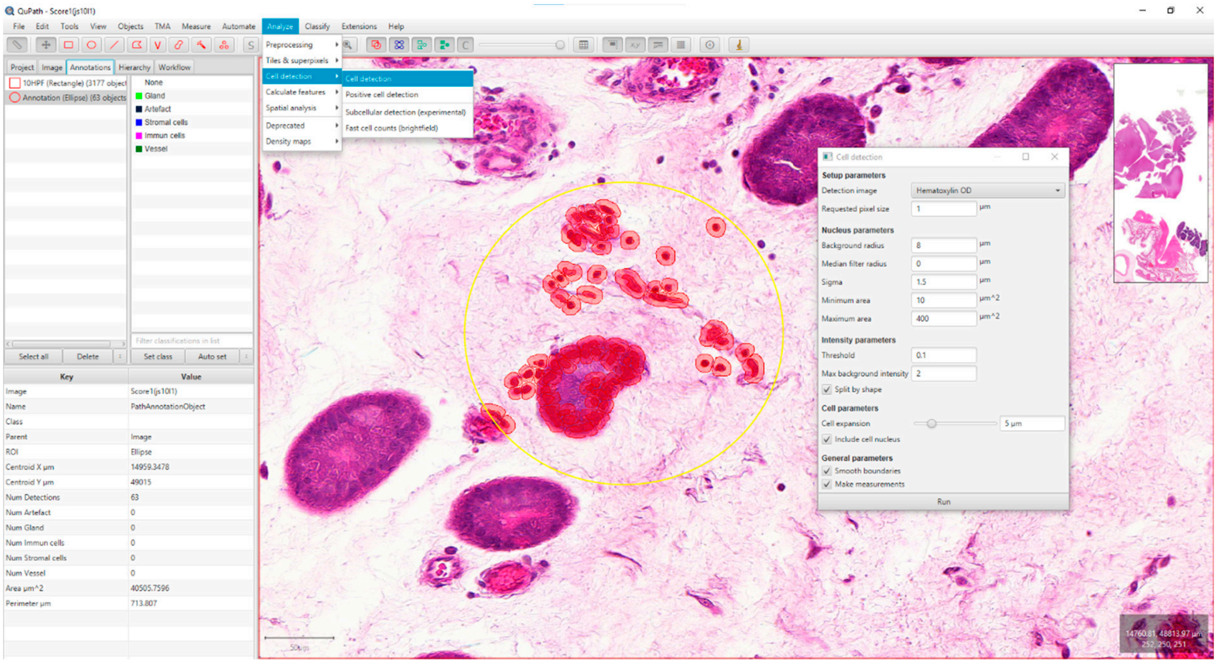

Cell detection command in a random area for demonstration.

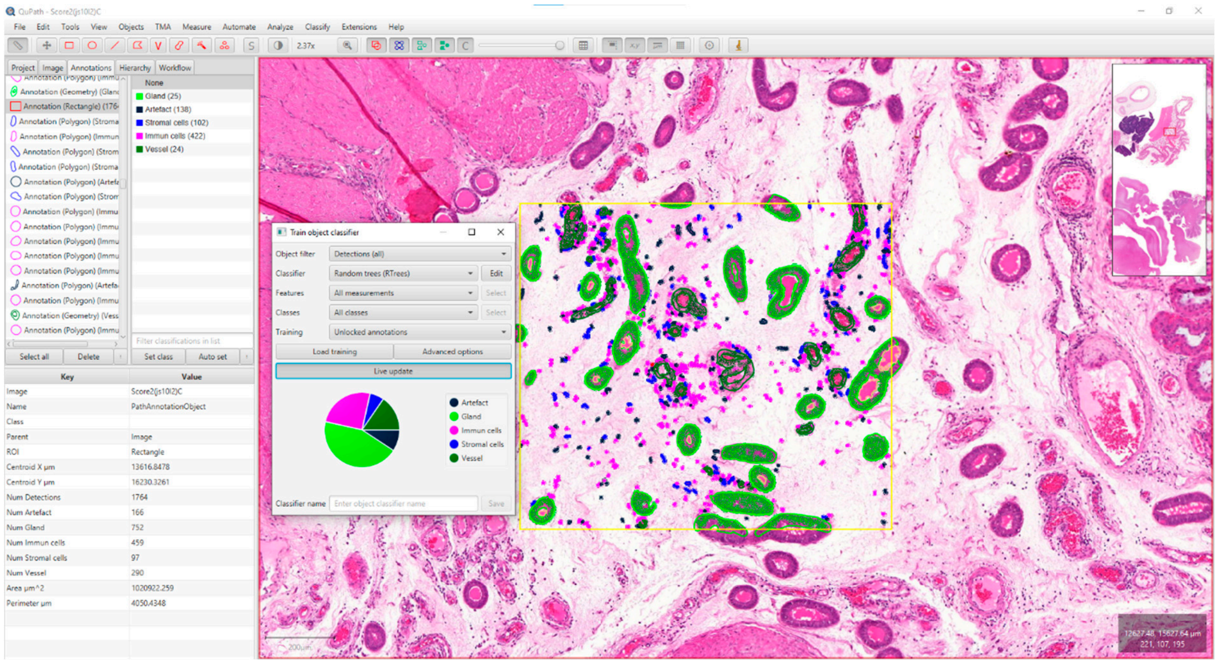

The training of a random trees classifier with live update.

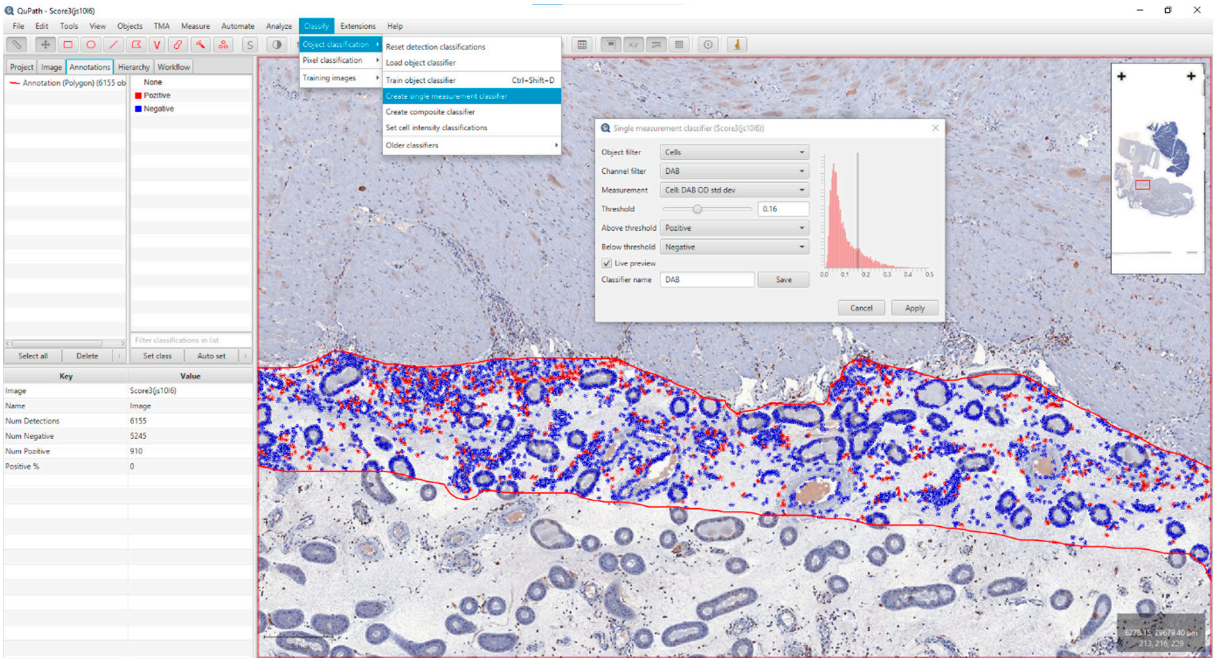

The training of a single measurement classifier with live preview.

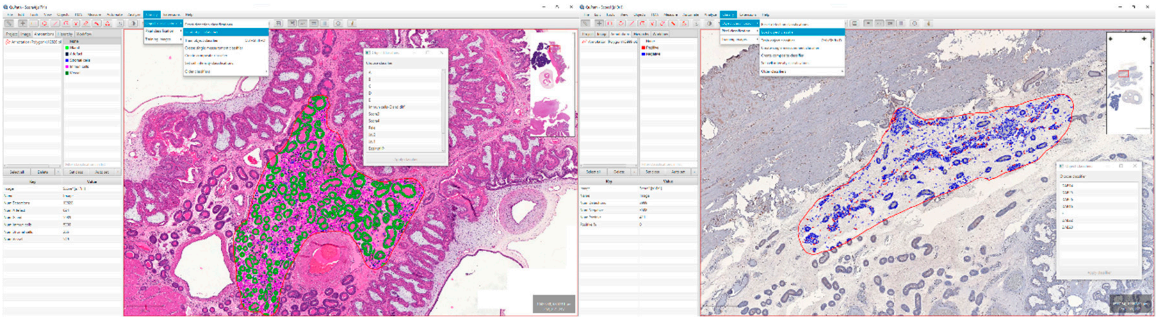

The selection of the appropriate classifier.

The morphological features, computed for each cell and used as input for classification

1. Nucleus: Area
2. Nucleus: Perimeter
3. Nucleus: Circularity
4. Nucleus: Max caliper
5. Nucleus: Min caliper
6. Nucleus: Eccentricity
7. Nucleus: Hematoxylin OD mean
8. Nucleus: Hematoxylin OD sum
9. Nucleus: Hematoxylin OD std dev
10. Nucleus: Hematoxylin OD max
11. Nucleus: Hematoxylin OD min
12. Nucleus: Hematoxylin OD range
13. Nucleus: Eosin OD mean
14. Nucleus: Eosin OD sum
15. Nucleus: Eosin OD std dev
16. Nucleus: Eosin OD max

17. Nucleus: Eosin OD min
18. Nucleus: Eosin OD range
19. Cell: Area
20. Cell: Perimeter
21. Cell: Circularity
22. Cell: Max caliper
23. Cell: Min caliper
24. Cell: Eccentricity
25. Cell: Hematoxylin OD mean
26. Cell: Hematoxylin OD std dev
27. Cell: Hematoxylin OD max
28. Cell: Hematoxylin OD min
29. Cell: Eosin OD mean
30. Cell: Eosin OD std dev
31. Cell: Eosin OD max
32. Cell: Eosin OD min
33. Cytoplasm: Hematoxylin OD mean
34. Cytoplasm: Hematoxylin OD std dev
35. Cytoplasm: Hematoxylin OD max
36. Cytoplasm: Hematoxylin OD min
37. Cytoplasm: Eosin OD mean
38. Cytoplasm: Eosin OD std dev
39. Cytoplasm: Eosin OD max
40. Cytoplasm: Eosin OD min
41. Nucleus/Cell area ratio
